# Development of 3D-Printed Bicompartmental Devices by Dual-Nozzle Fused Deposition Modeling (FDM) for Colon-Specific Drug Delivery

**DOI:** 10.3390/pharmaceutics15092362

**Published:** 2023-09-21

**Authors:** Fatemeh Shojaie, Carmen Ferrero, Isidoro Caraballo

**Affiliations:** Departamento Farmacia y Tecnología Farmacéutica, Facultad de Farmacia, Universidad de Sevilla, C/Prof. García González No. 2, 41012 Sevilla, Spain; fatsho@alum.us.es (F.S.); caraballo@us.es (I.C.)

**Keywords:** 3D printing, dual-nozzle fused deposition modeling, 5-aminosalicylic acid, bicompartmental devices, colon-targeted drug delivery

## Abstract

Dual-nozzle fused deposition modeling (FDM) is a 3D printing technique that allows for the simultaneous printing of two polymeric filaments and the design of complex geometries. Hence, hybrid formulations and structurally different sections can be combined into the same dosage form to achieve customized drug release kinetics. The objective of this study was to develop a novel bicompartmental device by dual-nozzle FDM for colon-specific drug delivery. Hydroxypropylmethylcellulose acetate succinate (HPMCAS) and polyvinyl alcohol (PVA) were selected as matrix-forming polymers of the outer pH-dependent and the inner water-soluble compartments, respectively. 5-Aminosalicylic acid (5-ASA) was selected as the model drug. Drug-free HPMCAS and drug-loaded PVA filaments suitable for FDM were extruded, and their properties were assessed by thermal, X-ray diffraction, microscopy, and texture analysis techniques. 5-ASA (20% *w*/*w*) remained mostly crystalline in the PVA matrix. Filaments were successfully printed into bicompartmental devices combining an outer cylindrical compartment and an inner spiral-shaped compartment that communicates with the external media through an opening. Scanning electron microscopy and X-ray tomography analysis were performed to guarantee the quality of the 3D-printed devices. In vitro drug release tests demonstrated a pH-responsive biphasic release pattern: a slow and sustained release period (pH values of 1.2 and 6.8) controlled by drug diffusion followed by a faster drug release phase (pH 7.4) governed by polymer relaxation/erosion. Overall, this research demonstrates the feasibility of the dual-nozzle FDM technique to obtain an innovative 3D-printed bicompartmental device for targeting 5-ASA to the colon.

## 1. Introduction

Over the last several decades, three-dimensional (3D) printing has emerged as a groundbreaking technology, revolutionizing several industries, including the healthcare sector. The ability to fabricate intricate structures layer-by-layer under computer-aided software control has opened new possibilities in the design of implants and prostheses, anatomic models, surgery tools, and pharmaceuticals, among other applications [1,2,3]. In the pharmaceutical field, 3D printing has the potential to manufacture on-demand and personalized dosage forms tailored to individual patient’s needs. This customization holds the promise of improving patient compliance and treatment efficacy. Additionally, 3D printing offers greater flexibility compared to conventional pharmaceutical processes, enabling the precise control of drug dose and distribution and the design of dosage forms with multiple drugs, complex geometries, and modulated drug release kinetics [1,4,5,6,7].

Among the various 3D printing techniques applied in the pharmaceutical sector, fused deposition modeling (FDM) is the most widely researched due to its simplicity, versatility, and cost-effectiveness. The number of patent applications involving this technology has increased sharply, with more than 700 published European patent applications over the last 20 years [8]. FDM is a thermal extrusion technique based on the deposition of molten thermoplastic filaments in sequential layers onto a building platform to print a 3D solid structure according to a computer-aided design (CAD) model. The diameter and mechanical properties of filaments and printing parameters, such as the nozzle and platform temperatures, flow rate, printing speed, layer height, and infill pattern and density, among others, should be carefully controlled to achieve a high-quality 3D-printed device [7,9,10,11]. Preparation of the filaments used as feedstock materials in FDM printers is usually carried out by hot-melt extrusion (HME). During this process, formulations containing the matrix-forming polymer, drugs, and, if necessary, other excipients are homogeneously mixed, heated, and extruded through a die into filaments with a suitable diameter. The coupling of HME and FDM technologies expands the number of pharmaceutical-grade polymers processable by 3D printing, and hence, possibilities for the design of drug delivery systems [1,4,12,13,14].

FDM printers equipped with two printheads (dual-nozzle) allow for the simultaneous extrusion of two different polymeric filaments to produce a single structure. Thus, even more sophisticated dosage forms able to control drug release can be developed [15,16,17]. Different studies have been reported in the literature dealing with the application of the dual-nozzle FDM technique to fabricate multicompartment drug delivery systems, mostly in the form of bi- or multilayer [14,15,17,18,19,20,21] and shell-core [9,16,17,18,22,23] designs. The combination of hybrid formulations and/or structurally different sections into the same dosage form opens new opportunities to achieve customized drug release profiles [1].

In the present study, a novel bicompartmental device intended for colon-specific drug delivery is introduced, combining different formulations and geometries for each compartment. Regarding the formulation, hydroxypropylmethylcellulose acetate succinate (AQOAT^®^ HPMCAS-HG) and polyvinyl alcohol (Parteck^®^ MXP PVA) were selected as the matrix-forming polymers of the external pH-dependent and the drug-containing internal water-soluble compartments, respectively. Hydroxypropylmethylcellulose acetate succinate (HPMCAS) is a mixture of acetic acid and monosuccinic acid esters of hydroxypropylmethyl cellulose, available in several grades with different particle sizes and chemical substitution levels. HPMCAS-HG represents a granular grade with the highest ratio between acetyl and succinoyl groups, with its pH threshold for solubilization (pH ≥ 6.5) being the highest among all HPMCAS grades. Thus, HPMCAS-HG is usually considered for the formulation of colon-targeted dosage forms [24,25,26]. Due to their thermoplastic properties, HPMCAS derivatives have proven to be suitable for the manufacture of delayed and sustained release devices by FDM, after optimization of the formulation by the addition of plasticizers and/or other additives [13,21,27,28,29,30,31].

Polyvinyl alcohol (PVA) is a copolymer of vinyl acetate and vinyl alcohol that exhibits desirable properties for pharmaceutical use, including hydrophilicity, non-toxicity, non-carcinogenicity, and biodegradability [24,32]. The relatively modern grade of Parteck^®^ MXP 4–88 PVA presents a special particle size specifically developed for HME applications. This thermoplastic polymer is compatible with a wide variety of APIs, facilitating stable formulations and enabling the achievement of high drug loads [33]. PVA has been extensively employed in the production of drug-loaded filaments by HME with the addition of suitable plasticizers to improve its processability. These filaments have been used as feedstock materials for the manufacture of immediate- or sustained-release dosage forms by FDM [17,34,35,36,37,38].

5-Aminosalicylic acid (5-ASA), also known as mesalazine or mesalamine, was chosen as an ideal model drug to test the efficiency of the bicompartmental device in targeted and controlled drug release. Due to its anti-inflammatory effects, 5-ASA is widely recognized as a primary treatment option for inflammatory bowel disease (IBD), also having a protective effect against colorectal cancer. Because of its topical mode of action and substantial absorption in the upper intestinal region, the targeted delivery of 5-ASA to the colon is crucial for optimal therapeutic outcomes [39,40,41,42]. Oral pH-dependent dosage forms are an interesting approach for this drug, as they remain intact in the stomach and the small intestine, promoting the release of 5-ASA in the higher-pH colonic environment [39,41]. To the best of our knowledge, only one report in the literature [43] has dealt with the FDM 3D printing of oral drug delivery systems for colon-targeting 5-ASA. However, the authors achieved a reduced drug loading (0.06% *w*/*w*) because they incorporated the drug by diffusion into commercial PVA filaments.

Taking advantage of the benefits of the dual-nozzle FDM technique, the main purpose and novelty of the present investigation is the design and manufacture of 3D-printed bicompartmental devices with hybrid formulations and geometries for the colon-specific drug delivery of 5-ASA. To achieve this main goal, made-in-house filaments were obtained using single-screw extruders from optimized drug-free HPMCAS-HG and drug-loaded (20% *w*/*w*) PVA formulations. By the simultaneous FDM printing of both filaments, an innovative and challenging 3D-printed structure was obtained, consisting of a pH-dependent HPMCAS-based external compartment of cylindrical geometry and a water-soluble PVA-based internal compartment containing the drug. This inner compartment has a spiral shape and communicates with the surrounding media through an opening gap on top of the device. We hypothesized that the intricate geometry of the inner compartment along with the pH dependence of the outer compartment will allow for the incorporation of a relatively high drug load and achievement of a delayed and controlled drug release.

## 2. Materials and Methods

### 2.1. Materials

Hydroxypropylmethylcellulose acetate succinate (HPMCAS) AQOAT^®^ granular-grade AS-HG (22–26% methoxy content, 6–10% hydroxypropoxy content, 10–14% acetyl content, 4–8% succinoyl content) was supplied by Shin–Etsu Chemical (Tokyo, Japan) (batch 8053093). This polymeric cellulose derivative is insoluble in acidic media but swells and dissolves quickly in alkaline conditions (pH ≥ 6.5). Polyvinyl alcohol (PVA) (Parteck^®^ MXP) was purchased from Merck KGaA (Darmstadt, Germany) (batch F2158464 132). This water-soluble polymer is milled polyvinyl alcohol (PVA 4-88) with a special particle size, specifically developed for HME. Triethyl citrate (TEC) (Sigma-Aldrich Chemie, Steinheim, Germany) (batch STBJ1790) and sorbitol (Parteck^®^ SI 150) (Merck-KGaA, Darmstadt, Germany) (batch MP20027683 048) were used as plasticizers to facilitate the extrusion process. 5-Aminosalicylic acid (5-ASA, mesalazine, or mesalamine) (batch 191016-J-1) was supplied by Acofarma (Barcelona, Spain) and selected as the model drug. Magnesium stearate (MgS) (batch 216188) and colloidal silicon dioxide (Aerosil^®^ 200) (batch 217040) were also acquired from Acofarma (Barcelona, Spain) and used as a lubricant and glidant, respectively.

Polylactic acid (PLA), a commercially available filament, was purchased from Raise3D^TM^ (Irvine, CA, USA) (batch 180802202) to use, when necessary, as a support material in the 3D printing process.

All other solvents and chemical reagents were of analytical grade.

### 2.2. Preparation of HPMCAS and Drug-Loaded PVA Filaments

To optimize the formulation of the HPMCAS-HG and drug-loaded PVA filaments, preliminary studies were performed using plasticizers and other additives in the following concentration ranges (based on the total weight): TEC (10–20% *w*/*w*), sorbitol (10–30% *w*/*w*), MgS (1–2% *w*/*w*), and Aerosil^®^ (1–2% *w*/*w*). After optimization, the final compositions of the made-in-house filaments used in this study are presented in Table 1. For the HPMCAS-HG filament preparation, the polymer was previously dried in an oven (50 °C, 2 h) and premixed with the TEC plasticizer using a mortar and pestle, followed by 5 min of mixing at 34 rpm in a Turbula^®^ T2F blender (Willy A. Bachofen, Basel, Switzerland). This mixture was kept overnight for the migration of the TEC. After the pre-plasticization of the polymer, the magnesium stearate lubricant was added, and it was mixed for 5 min more. The final mixture was subsequently extruded by a single-screw extruder, Noztek Touch (Noztek, Shoreham, West Sussex, UK), with a nozzle diameter of 1.75 mm and equipped with a 33 rpm 9 Nm motor. The blend (20 g) was extruded at an angle of 45° and a feeding rate of 5 g/min, with the fan on, a screw speed of 20 rpm, and a temperature of 150 °C.

For the drug-loaded filament preparation, the PVA polymer, the sorbitol plasticizer, and the 5-ASA drug were dried in an oven at 70 °C for 2 h and directly mixed in the Turbula^®^ T2F blender (Willy A. Bachofen, Basel, Switzerland) for 15 min at 34 rpm. After the addition of Aerosil^®^, the mixing was continued for a further 5 min. The physical mixture (20 g) was fed into a single-screw extruder Noztek Touch (Noztek, Shoreham, West Sussex, UK), with a 1.75 mm nozzle diameter, and a 53 rpm 6 Nm motor. The extrusion process was performed at an angle of 45° and a feeding rate of 2 g/min, with the fan on, a screw speed of 30 rpm, and a temperature of 180 °C.

Extrusion parameters were in the range of those reported in the literature for filament formulations containing HPMCAS and PVA polymers [24,26,27,34,36,37] and were carefully controlled to ensure the reproducibility and quality of the filaments.

Since the uniformity of the diameter is essential to guarantee consistent printing, the diameters of the extruded filaments were measured (every 10 cm) using a digital micrometer (Comecta, SA, Barcelona, Spain).

The prepared filaments were stored at room temperature in sealed plastic bags with desiccant to prevent moisture absorption.

### 2.3. Solid State Characterization and Morphological Evaluation

#### 2.3.1. Differential Scanning Calorimetry (DSC)

Differential scanning calorimetry (DSC) was employed to analyze the thermal properties of the raw materials, physical mixtures, and extruded filaments. DSC measurements were performed with DSC Q20 equipment (TA Instruments, New Castle, DE, USA), available at Centro de Investigación, Tecnología e Innovación (CITIUS) (Universidad de Sevilla). Accurately weighed samples (2–8 mg and 26 mg for the solid and liquid samples, respectively) were hermetically sealed in Tzero aluminum pans (40 μL) and heated up to 350 °C at a heating rate of 10 °C/min. Nitrogen was used as an inert purge gas with a flow rate of 100 mL/min. Indium and bismuth were used as standards to calibrate the DSC instrument. The data were analyzed using Universal Analysis 2000 V4.5A software (TA Instruments, New Castle, DE, USA).

#### 2.3.2. Thermogravimetric Analysis (TGA)

Thermogravimetric analysis (TGA) was performed to test the moisture content and to assess the degradation profile of the raw materials, physical mixtures, and extruded filaments. TGA measurements were conducted on SDT Q600 equipment (TA Instruments, New Castle, DE, USA), also available at CITIUS. Accurately weighed samples (2–9 mg and 11 mg for the solid and liquid samples, respectively) were introduced to open alumina crucibles (90 μL) and heated to 800 °C at a heating rate of 10 °C/min. The measurements were conducted under a dynamic nitrogen atmosphere (100 mL/min). The calibration materials for the TGA instrument were bismuth and zinc. The data were analyzed using Universal Analysis 2000 V4.5A software (TA Instruments, New Castle, DE, USA).

#### 2.3.3. X-ray Powder Diffraction (XRPD)

X-ray powder diffraction measurements were conducted to monitor the physical state transformation of the materials due to the thermal treatment of the extrusion process. The XRPD patterns of the raw materials, physical mixtures, and extruded filaments were obtained using a Bruker D8 Advance A25 diffractometer (Bruker, Karlsruhe, Germany) with Ni-filtered CuKα radiation (λ 1.5418 Å) and a LynxEye^TM^ detector (CITIUS, Universidad de Sevilla). The X-ray generator power was set to 40 kV and 30 mA. The scanning range was from 3° to 70° (2θ), with a step size of 0.015° and a step time of 0.1 s.

#### 2.3.4. Scanning Electron Microscopy (SEM)

The surface morphologies of the raw materials, physical mixtures, extruded filaments, and 3D-printed devices were evaluated by the Microscopy Service of CITIUS (Universidad de Sevilla) using a high-resolution scanning electron microscope (FEGSEM) FEI TENEO (FEI Company, Hillsboro, OR, USA), operating at 5 kV and 0.40 nA. Prior to imaging, the samples were mounted onto aluminum stubs using carbon double adhesive tape (Nisshin Em, Shanghai, China) and then coated with a 10 nm-thin Pt layer using a Leica EM ACE600 high vacuum sputter coater (Leica Microsystems, Vienna, Austria). Low- and high-magnification images were acquired.

### 2.4. Mechanical Characterization of Filaments

The most critical mechanical properties to determine the suitability of filaments for 3D printing are their flexibility, brittleness, and stiffness. A TA.XTPlus texture analyzer (Stable Micro Systems Ltd., Godalming, Surrey, UK) was used to evaluate the mechanical properties of the extruded filaments. The flexibility and brittleness were measured using the three-point bending (3PB) test adapted from Xu et al. [44]. The texture analyzer was equipped with a 3PB rig with thin blades (HDP/3PB-type). Homogenized extruded filaments for each formulation (*n* = 6) were cut into 6 cm segments and placed on the sample holder with a 30 mm supporting gap. The trigger force and test speed were set to 4.2 g and 10 mm/s, respectively. The maximum distance below the supported sample, i.e., the fracture distance, was set to 10 mm. The maximum force and fracture distance data were recorded and analyzed using Exponent software version 6.1.20.0 (Stable Micro Systems Ltd., Godalming, Surrey, UK). The area under the curve (AUC) and maximum stress of the stress (kg/mm^2^)–strain (%) plots were calculated using the Macro program of the software Exponent software version 6.1.20.0.

The stiffness test was also adapted from Xu et al. [44] and carried out using an HDP/BS probe set and a flat solid metal surface as the sample holder. The filament samples (*n* = 6) were cut into 6 cm segments and placed on top of the platform. The trigger force was set to 50 g, and the blade was set to cut the filament until 1 mm in distance (57% strain) at a speed of 2 mm/s. The data collection and analysis were also performed using the Exponent software version 6.1.20.0.

### 2.5. Filament Drug Content Determination

The drug contents of the PVA filaments (*n* = 3) were determined by placing homogenized and accurately weighed sections (~210 mg) of the drug-loaded filaments into 500 mL of phosphate buffer media (pH 7.4) under agitation at 50 rpm until complete dissolution. The filtered samples (5 mL) were removed from the dissolution vessel and analyzed by UV-Vis spectrophotometry (Agilent 8453, Waldbronn, Germany) at λ 332 nm. The 5-ASA concentration was determined using an appropriate reference curve (C (mg/mL) = −0.00057 + 0.04642 × A, r^2^ = 0.9992).

### 2.6. Design and Elaboration of 3D-Printed Systems

**Figure 1:**
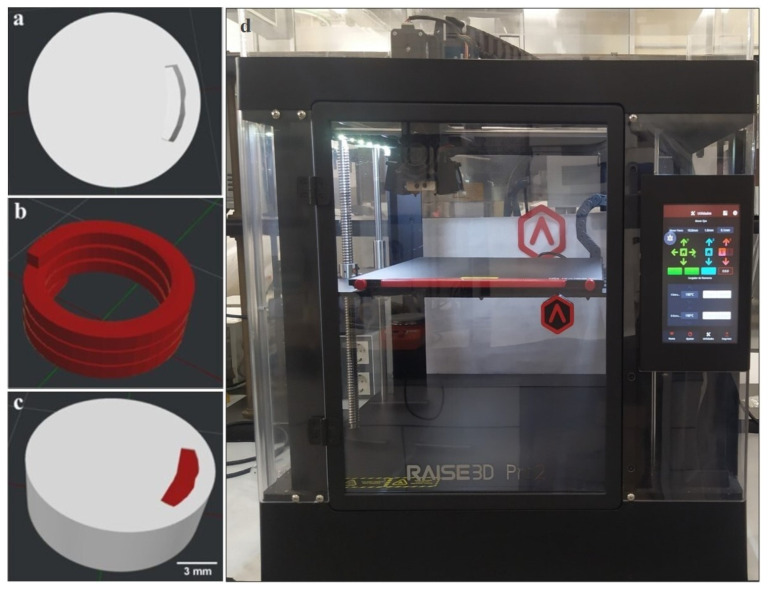
Digital designs (IdeaMaker software version 3.1.7) of (**a**) cylindrical HPMCAS-based external compartment (top view), (**b**) drug-loaded PVA-based internal compartment (spiral shape), (**c**) bicompartmental device for FDM printing using a Pro2 Dual Extruder Raise3D printer (**d**).

The 3D-printed model was designed using BlocksCAD software version 1.13.0 (BlocksCAD, Burlington, MA, USA). The design was then exported as a stereolithographic (.stl) file and sliced using IdeaMaker version 3.1.7 software (Raise3D Technologies, Irvine, CA, USA) to generate the G-code for printing. The extruded filaments were printed using a Pro2 Dual Extruder Raise3D printer (Raise3D Technologies, Irvine, CA, USA), an FDM printer supplied with two printheads (0.4 mm nozzles) (Figure 1). Three-dimensional-printed bicompartmental devices with a cylindrical shape (14 mm diameter × 5 mm height) were prepared using the HPMCAS-HG filament to print the external pH-dependent compartment (white color in Figure 1) and the drug-loaded PVA filament to print the internal soluble compartment, a channel with a spiral shape (red color in Figure 1). This compartment communicates with the surrounding media through an opening gap on top of the device with a small size (1 mm width × 5 mm length) to achieve a slow water uptake through the inner structure. The printing temperatures were 195 °C for the HPMCAS-HG filament and 190 °C for the drug-loaded PVA filament. The rest of the processing parameters are summarized in Table 2 and in the range of those reported in the literature for 3D-printed devices made from HPMCAS or PVA polymers [17,24,27,31,36].

For comparison purposes, the internal spiral compartment from the drug-loaded PVA filament was also printed using the same printer and the printing settings specified in Table 2. A support of commercial polylactic acid (PLA) was used in this case to prevent structural collapse and ensure successful layer deposition.

To prevent moisture absorption, drug-loaded filaments were kept in the oven (70 °C) the day before printing, as the quality of the extruded filament greatly influenced the final properties of the 3D-printed system.

To evaluate the consistency of the 3D printing process, the weight uniformity of the 3D-printed bicompartmental devices (*n* = 6) was determined using a high-precision analytical balance (Shimadzu AUW120, Manila, Philippines), and their dimensions (*n* = 6) were measured using an electronic micrometer (Comecta, SA, Barcelona, Spain).

### 2.7. X-ray Microcomputer Tomography (XµCT) of 3D-Printed Devices

X-ray microcomputer tomography was conducted to visualize the external and internal microstructure of the 3D-printed bicompartmental devices in 3D. X-ray tomography images were acquired using the Zeiss Xradia 610 Versa equipment (Zeiss, Jena, Germany) available at CITIUS (Universidad de Sevilla). The scanning procedure was conducted at a voltage of 40 kV. The 3D-printed structure was scanned using no filter, an optical magnification of 0.4×, and a pixel size of 16.5 µm. The exposure time per frame was 3 s. The tomograms were reconstructed from the raw data using Reconstructor Scout-and-Scan v. 16.0 software (Zeiss, Jena, Germany) and exported as a 16-bit TIFF file for visualization and analysis.

### 2.8. In Vitro Drug Release Studies of 3D-Printed Systems

The studies of the in vitro drug release from 3D-printed bicompartmental devices (*n* = 3) were conducted using a USP Apparatus II (Erweka DT 600 HH, Heusenstamm, Germany) with a paddle rotation speed of 50 rpm. The experiments were run at 37 ± 0.5 °C using the following biorelevant dissolution media (500 mL) and residence times (sequential pH change method): simulated gastric fluid (SGF) (without pepsin; pH 1.2) for 2 h, simulated intestinal fluid (SIF) (without pancreatin; pH 6.8) for an additional 2 h, and phosphate buffer (pH 7.4) until the end of the study (24 h). Dissolution media were prepared according to USP standards. Samples (5 mL) were withdrawn at predetermined time intervals, filtered (0.45 μm), and analyzed using UV–Vis spectrophotometry (Agilent 8453, Waldbronn, Germany) at λ 302 nm (pH 1.2), 330 nm (pH 6.8), and 332 nm (pH 7.4). The concentrations of the released drug were determined using standard calibration curves (r^2^ = 0.9970–0.9999). Cumulative corrections were made for the previously removed samples when determining the total amount released.

For comparative purposes, the drug release from the internal spiral compartment (*n* = 3) was also tested using the same procedure as for the 3D-printed bicompartmental devices.

The drug release kinetics of the 3D-printed bicompartmental devices were investigated using different mathematical models: the Higuchi (1), Korsmeyer–Peppas (2), Peppas–Sahlin (3), and zero-order (4) equations [45,46].

(1) $$\frac{M_{t}}{M_{\infty }}=k_{H}\cdot t^{0.5}$$

(2) $$\frac{M_{t}}{M_{\infty }}=k_{K}\cdot t^{n}$$

(3) $$\frac{M_{t}}{M_{\infty }}=k_{d}\cdot t^{m}+k_{r}\cdot t^{2m}$$

(4) $$\frac{M_{t}}{M_{\infty }}=k_{0}\cdot t$$

where *M_t_*/*M_∞_* is the fractional drug release at time *t* (*M_∞_* is considered equivalent to the drug loading); *k_H_* is the Higuchi kinetic constant; *k_K_* is the Korsmeyer kinetic constant; *n* is a release exponent that depends on the release mechanism and the geometry of the system; *k_d_* and *k_r_* are the diffusion and relaxation rate constants, respectively; *m* is the purely Fickian diffusion exponent for a device of any geometrical shape that exhibits controlled release, and *k_0_* is the zero-order release rate constant.

The optimum values for the parameters in each equation were determined by linear or non-linear least-squares fitting methods using SPSS^®^ Statistics 26 software (IBM Corp., Armonk, NY, USA). The adjusted coefficient of determination (r^2^_adj_) and F-ratio probability were used as the criteria to evaluate the degree of fit of the different mathematical models.

## 3. Results and Discussion

### 3.1. Preparation of HPMCAS and Drug-Loaded PVA Filaments

The aim of the extrusion process was to obtain both HPMCAS-HG and drug-loaded PVA filaments suitable for FDM 3D printing. As outlined in Section 2.2., preliminary studies were performed to screen the optimum ratio of the components (polymer, plasticizer, drug, and auxiliary excipients) in each filament formulation. HPMCAS-HG (88% *w*/*w*) was selected as an enteric polymer (pH threshold 6.5) for the first filament formulation, and TEC (10% *w*/*w*) was added as a liquid plasticizer to lower the processing temperatures [13,24,47]. To promote the molecular interaction between the dry polymer and the plasticizer, a pre-plasticization step was carried out by homogeneously mixing the HPMCAS-HG and TEC before adding the other additives. Magnesium stearate (2% *w*/*w*) was used as a lubricant to facilitate the homogenous transit of the powder mixture through the extruder [24,27].

For the second filament formulation, pharmaceutical-grade Parteck^®^ MXP PVA (51% *w*/*w*) was selected as an appropriate hydrophilic matrix-forming polymer for the preparation of the drug-loaded filament. Due to the high melt viscosity and melting point of neat PVA, Parteck^®^ SI 150 sorbitol (27% *w*/*w*) was used as a water-soluble solid plasticizer, as it has been demonstrated that it can form hydrogen bonds with PVA, reducing its glass transition temperature (T_g_) and facilitating thermal processing [36,37]. The impact of sorbitol on PVA was found to be dependent on its concentration, as more interactions can be established between the OH groups of both components, leading to a disruption of the structural regularity of PVA [32]. 5-ASA (20% *w*/*w*) was selected as the model drug due to its use as first-line therapy in the treatment of inflammatory bowel disease (IBD). This drug would benefit from the 3D printing design presented in this study to obtain colon-specific drug delivery. Moreover, 5-ASA can be processed by hot-melt extrusion due to its high thermal stability [39,41,43]. The glidant Aerosil^®^ (2% *w*/*w*) was also incorporated into the formulation to reduce cohesion and interparticle friction, improving the mixture flowability and the feeding of the powder into the extruder [34,48].

**Figure 2:**
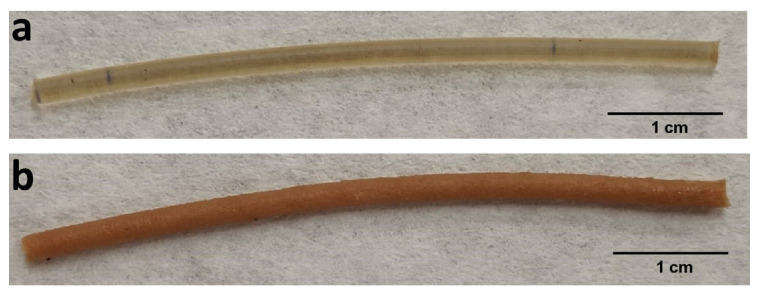
Digital pictures of (**a**) extruded HPMCAS-HG filament, (**b**) extruded drug-loaded PVA filament.

After the mixture preparation, the extrusion process of each formulation was carried out at different heating temperatures and screw rotation speeds to optimize the diameter and mechanical properties of the filaments. Table 3 summarizes the extrusion process parameters and diameters of the extruded filaments. As stated in Section 2.2, the optimal conditions for the HPMCAS-HG filament production were a heating temperature of 150 °C and a screw speed of 20 rpm. The extrusion process of the drug-loaded PVA filament was performed at 180 °C and 30 rpm. The flow speed was between 15 and 16 mm/min for both filaments, although the residence time was higher for the PVA filament, probably due to the higher friction caused by the presence of the drug [11]. Both the HPMCAS-HG and drug-loaded PVA filaments showed uniform and suitable diameters for FDM printing, ranging from 1.77 to 1.79 mm. Digital pictures of the filaments were also taken to comparatively evaluate their aspects. As depicted in Figure 2, the HMPCAS-HG filament was white to yellowish in color, translucent, and presented a smooth surface. Due to the presence of mesalazine (fairly pink color), the drug-loaded PVA filament was opaque, with a brownish color and a slightly rough surface.

### 3.2. Solid State Characterization and Morphological Evaluation

#### 3.2.1. Differential Scanning Calorimetry (DSC)

**Figure 3:**
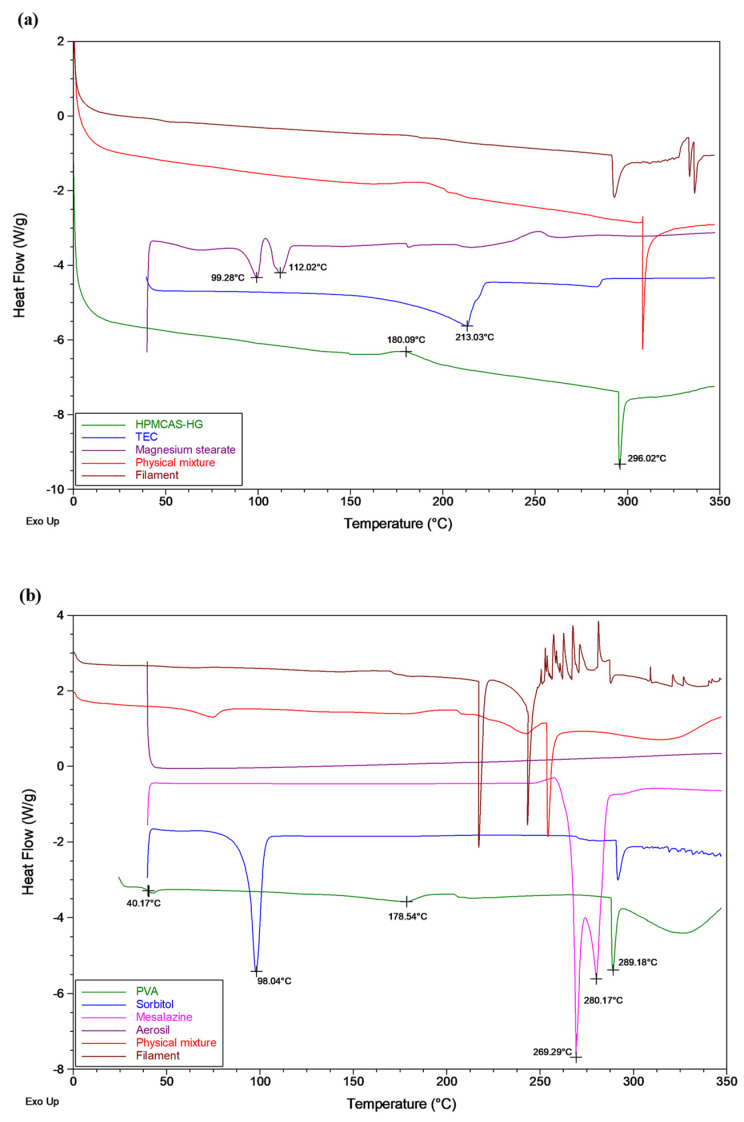
DSC thermograms of (**a**) HPMCAS-HG, TEC, magnesium stearate, and the corresponding physical mixture and extruded filament, (**b**) PVA, sorbitol, mesalazine, Aerosil^®^, and the corresponding physical mixture and extruded filament.

DSC was conducted to investigate the thermal properties of the raw materials, physical mixtures, and extruded filaments. HPMCAS-HG is an amorphous polymer that, according to the manufacturer [25], has a glass transition temperature (T_g_) of 122 °C. The DSC thermogram in Figure 3a also shows an exothermic peak at 180 °C, indicating crystallization, followed by a degradation endothermic peak at 296 °C. The TEC liquid plasticizer exhibited a boiling point of 213 °C. Magnesium stearate showed two endothermic peaks, at 99 °C and 112 °C, which correspond to water evaporation and melting of the lubricant, respectively. The results obtained for the raw materials agree with those of previous studies [13,30,49]. The DSC thermogram of the physical mixture was similar to that of HPMCAS-HG, the main component in the mixture. However, the exothermic peak corresponding to the crystallization of the polymer disappeared in the DSC curve of the filament, confirming the complete conversion of the polymer to the amorphous state.

As shown in Figure 3b, PVA is a semi-crystalline polymer, which showed an apparent T_g_ at 40 °C, a slight signal drop in the range of 90–140 °C, associated with the gradual loss of water bound to the polymer, a broad endothermic peak at 178 °C, indicative of the melting temperature (T_m_), and a degradation endothermic peak at 289 °C. De Jaeghere et al. [32] also found two endothermic signals corresponding to water evaporation and melting of the polymer for PVA grades with a high degree of hydrolysis (>70%), in contrast to those with a low degree of hydrolysis (<50%), where only water evaporation was observed. Hence, PVA 4-88 (approximately 88% hydrolyzed) presented a certain degree of crystallinity due to the increased number of OH groups able to form hydrogen bonds. The sorbitol solid plasticizer showed a melting endotherm at 98 °C. The crystalline 5-ASA drug exhibited a double melting peak that could be attributed to different crystal sizes [50]. The first endotherm (269 °C) corresponded to the melting of smaller crystals while the second one (280 °C) was characteristic of the melting of larger crystals. Only a weak and very broad endotherm (below 100 °C) could be found in the DSC curve of Aerosil^®^ due to the evaporation of adsorbed water. The thermal events of the raw materials agree with the data provided by the manufacturer [33] and the values reported in the literature [15,36,38,39,51,52]. The thermogram of the physical mixture resembled that of PVA (the main component), with the melting peaks of sorbitol and 5-ASA clearly distinguished, confirming that their solid-state structures were not modified by physical mixing. However, the melting endotherm of sorbitol completely disappeared in the DSC curve corresponding to the drug-loaded filament, as the extrusion process was carried out above its melting point. Hence, the sorbitol was gradually dissolved in the molten polymer. On the contrary, the 5-ASA remained crystalline in the filament, although a slight decrease in the melting temperature was noticed compared to the pure drug.

#### 3.2.2. Thermogravimetric Analysis (TGA)

**Figure 4:**
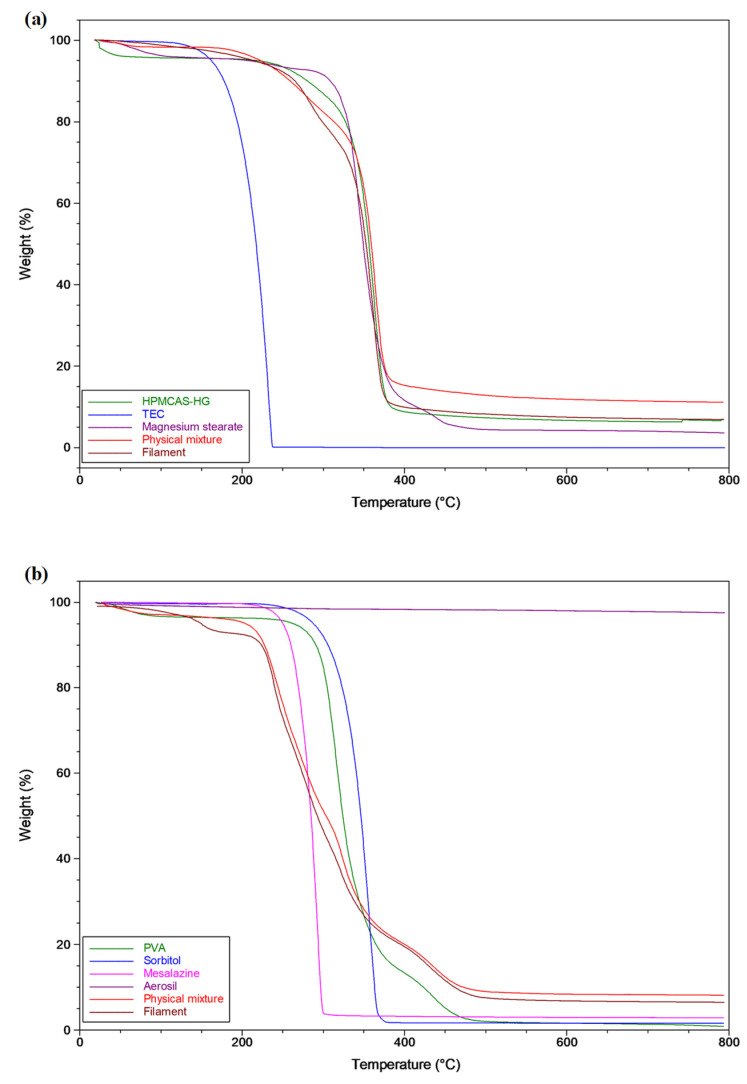
TGA thermograms of (**a**) HPMCAS-HG, TEC, magnesium stearate, and the corresponding physical mixture and extruded filament, (**b**) PVA, sorbitol, mesalazine, Aerosil^®^, and the corresponding physical mixture and extruded filament.

Thermogravimetric analysis (TGA) was used to examine the thermal stability of the raw materials, physical mixtures, and extruded filaments under the extrusion and 3D printing processing temperatures. The obtained TGA thermograms (Figure 4) demonstrate that all formulation components were thermally stable at the temperatures used for the extrusion (150 °C for the HPMCAS-HG filament and 180 °C for the drug-loaded PVA filament) and printing (195 °C for the HPMCAS-HG filament and 190 °C for the drug-loaded PVA filament) processes. The TGA data of the HPMCAS-HG polymer (Figure 4a) show a weight loss of about 3% *w*/*w* up to 100 ºC (water evaporation), but it remained stable up to 240 °C, with the maximum degradation temperature (T_max_) being 362 °C. Due to volatilization and decomposition, the decrease in the weight of the TEC started at 150 °C, reaching a maximum at 232 °C. The magnesium stearate showed a water loss of about 4% *w*/*w* and started to degrade at 300 °C (T_max_ 344 °C). These values agree with the data reported by other authors [13,27,30,39].

The mass loss observed in the PVA thermogram (Figure 4b) up to 100 °C was attributed to the loss of absorbed water (≈3% *w*/*w*), starting decomposition at 260 °C (T_max_ 316 °C). The sorbitol remained thermally stable until it was heated to ~250 °C, with its maximum degradation at 357 °C. The 5-ASA did not show signs of thermal decomposition until reaching approximately 230 °C (T_max_ 292 °C). These data are also in accordance with previous reports [15,39,52].

As illustrated in Figure 4a,b, the corresponding mixtures and filaments from both formulations did not exhibit significant weight loss below ~200 °C. Therefore, the TGA profiles demonstrate that the extruded filaments were suitable for 3D printing temperatures lower than 200 °C.

#### 3.2.3. X-ray Powder Diffraction (XRPD)

**Figure 5:**
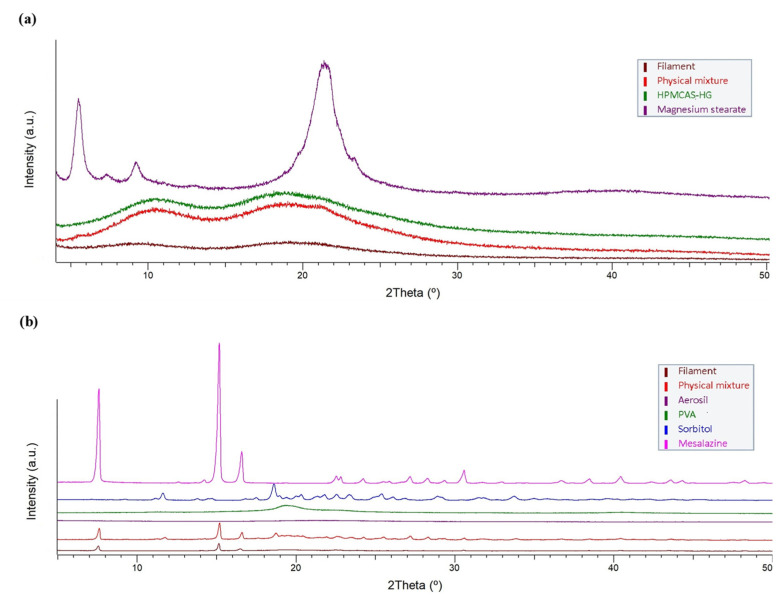
X-ray powder diffractograms of (**a**) HPMCAS-HG, magnesium stearate, and the corresponding physical mixture and extruded filament, (**b**) PVA, sorbitol, mesalazine, Aerosil^®^, and the corresponding physical mixture and extruded filament.

X-ray powder diffraction patterns of the raw materials, physical mixtures, and extruded filaments were collected (Figure 5) to qualitatively study the changes in the physical state of the materials. The XRPD pattern of HPMCAS-HG (Figure 5a) did not exhibit intensity diffraction peaks due to its amorphous state, in accordance with the DSC data (Figure 3a) and the XRD profiles reported in the literature [27,30]. Magnesium stearate presented typical peaks at 5.5° and 9.2° (2θ), which might be assigned to the anhydrate phase, as well as a large peak at 21.3° (2θ), which might be indicative of a hydrate phase [53]. This behavior agrees with the thermal analysis results, as a water loss event could be distinguished in the DSC scan (Figure 3a). The XRPD pattern of the physical mixture resembled that of the HPMCAS-HG polymer, the major component in the formulation. However, the XRPD pattern of the filament was attenuated, confirming the complete amorphization of the HPMCAS-HG and magnesium stearate during extrusion.

The XRPD pattern of the PVA polymer (Figure 5b), with a broad diffraction peak at 19.3° (2θ), confirms its semi-crystalline nature [34,36,37]. The sorbitol showed multiple sharp diffraction peaks, the most intense of which were at 11.6°, 18.6°, and 25.4° (2θ), representative of its crystalline structure [36,52]. The crystalline 5-ASA drug also exhibited intense diffraction peaks at 7.6, 15.2, and 16.6° (2θ), in agreement with the literature [39]. In contrast, the XRPD pattern of Aerosil^®^ confirms its amorphous character. The XRPD pattern of the physical mixture showed the characteristic peaks of PVA, sorbitol, and 5-ASA, although their intensities were attenuated according to their proportions in the mixture. The diffraction peak of the PVA became a very low-intensity diffused halo in the XRPD pattern of the filament, which also showed a complete absence of the crystalline peaks of the sorbitol, which is in good agreement with the disappearance of its melting endotherm in the DSC profile (Figure 3b). Only the 5-ASA remained crystalline in the PVA filament; however, its crystallinity was less intense after extrusion due to the partial solubilization of the 5-ASA in the amorphous PVA [39]. Overall, the X-ray powder diffraction data corroborate the DSC findings.

#### 3.2.4. Scanning Electron Microscopy (SEM)

**Figure 6:**
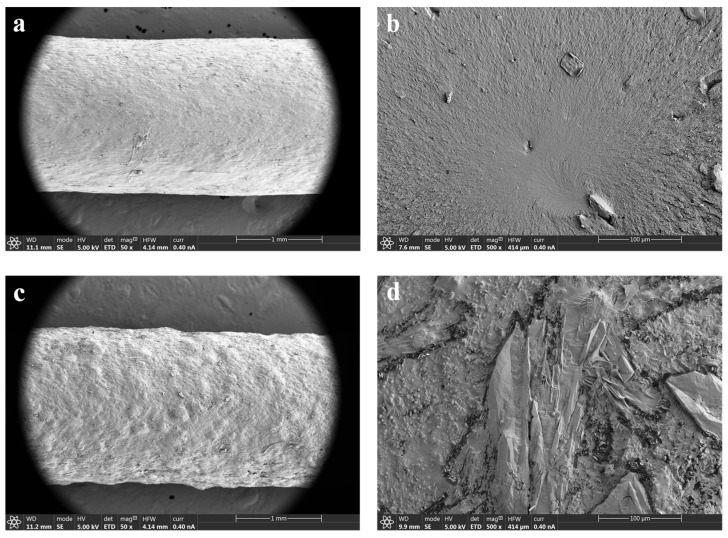
SEM microphotographs of (**a**) surface of HPMCAS-HG filament (50×); (**b**) cross-section of HPMCAS-HG filament (500×); (**c**) surface of drug-loaded PVA filament (50×); (**d**) cross-section of drug-loaded PVA filament (500×).

The surface morphology of raw materials, physical mixtures, and extruded filaments was investigated using SEM. A brief description of the morphology of the raw materials and their corresponding physical mixtures is included as Supplementary Information (Figure S1). Regarding the topography of the filaments, SEM images of the HPMCAS-HG extruded filament (Figure 6a,b) demonstrated a reasonably smooth surface, fine appearance, and a dense internal structure. Homogeneous extruded filaments with smooth surfaces are also reported in the literature for HPMCAS polymers, even in formulations containing drugs [21,30]. In contrast, the SEM images of the drug-loaded PVA filament (Figure 6c,d) show a filament also compact but with a certain degree of surface roughness due to the presence of 5-ASA. Crystalline drug particles with rod-like shapes dispersed throughout the filament can be clearly seen. These results are in accordance with the XRPD data, as crystalline peaks of 5-ASA can be observed in the diffractogram corresponding to the drug-loaded PVA filament (Figure 5b). As the extrusion was carried out at a significantly lower temperature than the melting point of 5-ASA (180 °C vs. 270 °C), crystalline drug domains remained within the filament. The negative impact of drug incorporation on the surface smoothness of filaments obtained from Parteck^®^ MXP PVA has also been described by other authors [19,54].

### 3.3. Mechanical Characterization of Filaments

During the 3D printing process, the extruded filaments are fed into the dual FDM 3D printer through a feeding gear system that drives them into the hot printing nozzles. Then, the filaments are fused and pushed out from the nozzles to be deposited layer-by-layer onto the printing platform. Therefore, suitable mechanical properties of the filaments are essential to ensure their feedability and/or printability [4,7,24,44,52]. Hence, in this study, the mechanical properties of the HPMCAS-HG and drug-loaded PVA filaments were investigated by texture analysis. Based on the quantitative method developed by Xu et al. [44], three-point bending (3PB) and stiffness tests were combined to calculate the parameters of flexibility, brittleness, and stiffness, the most determinant mechanical properties on filament processability [31,54].

**Figure 7:**
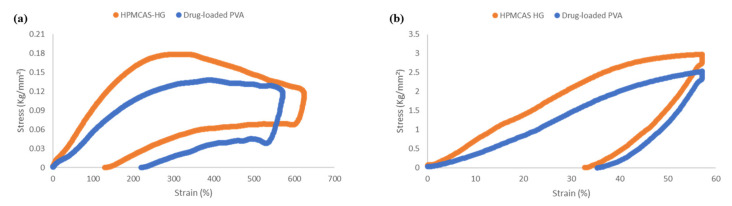
Representative stress–strain curves of three-point bending (3PB) (**a**) and stiffness (**b**) tests of HPMCAS-HG and drug-loaded PVA filaments.

The stress–strain curves (Figure 7) obtained from the 3PB and stiffness tests revealed that both the HPMCAS-HG and drug-loaded PVA filaments were flexible and did not break during testing (the stress applied on the filament bounced back after the set strain value and went back to zero). Considering that flexibility is the tolerance of the filament to bending without breaking [31], the strain (%) corresponding to the maximum stress (g/mm^2^) detected was used to determine the flexibility of the filaments. According to the data in Table 4, the drug-loaded PVA filament was slightly more flexible than the HPMCAS-HG filament. Good flexibility was also found by Crisan et al. [54] for PVA filaments containing different drug loads. On the other hand, Thakkar et al. [26] reported lower flexibility for HPMCAS-HG filaments compared to other HPMCAS grades (LG and MG). To determine the brittleness or the breaking of the filament without significant plastic deformation [31,36,55], the AUC in the 3PB stress–strain plot (Figure 7a) was calculated. Both filaments presented a limited degree of brittleness (Table 4), with the HPMCAS-HG filament being more ductile, and hence, less brittle, than the drug-loaded PVA filament. Therefore, the made-in-house filaments had enough mechanical strength to withstand the forces applied by the feeding gears without fracture. In agreement with other authors [36,44], the incorporation of the plasticizers (TEC or sorbitol) into the formulation of the filaments contributed to the improvement in their mechanical properties. The stiffness of the filaments represents the load required to achieve a certain deformation [31,55] (57% strain in this study) and was estimated as the AUC in the stiffness stress–strain plot (Figure 7b). According to the behavior reported by Xu et al. [44], the stiffness values were more reproducible than the brittleness measurements. The data in Table 4 show that the HPMCAS-HG filament had higher stiffness than the drug-loaded PVA filament. Zhang et al. [31] also developed HPMCAS-based filaments with good stiffness.

In summary, the made-in-house filaments exhibited adequate flexibility, mechanical strength, and stiffness, being suitable for feeding and 3D printing.

### 3.4. Filament Drug Content Determination

The drug content analysis of the drug-loaded PVA filaments was performed in phosphate buffer media (pH 7.4). After 60 min, the filaments were completely disintegrated and dissolved. The results obtained showed a 5-ASA loading of 99.7 ± 1.7%, in good agreement with the theoretical target value (42 mg of the drug for filament sections of 210 mg). An RSD lower than 2% indicates an acceptable drug content uniformity. These results further confirm the stability of the drug after the extrusion process, as also evidenced in the thermal analysis studies.

### 3.5. Three-Dimensional Printing and Physical Characterization of Three-Dimensional-Printed Systems

Using the made-in-house filaments as feedstock materials, 3D-printed bicompartmental devices were obtained by dual-nozzle FDM according to the design model in Figure 1. As illustrated, cylindrical devices (14 mm diameter × 5 mm height) were designed by combining an external pH-dependent compartment made from the HPMCAS-HG filament and an internal water-soluble compartment (spiral shape) made from the drug-loaded PVA filament. The internal channel communicates with the surrounding media through an opening gap on top of the device to control the water uptake and subsequent drug diffusion.

Prior to the dual-nozzle FDM printing process, preliminary studies were performed to optimize the printing temperatures for each filament and other printing parameters (Table 2) that could influence the quality of the 3D-printed systems. As outlined in the methodology (Section 2.6), the dual-nozzle FDM process was performed at optimized temperatures of 195 °C for the HPMCAS-HG filament and 190 °C for the drug-loaded PVA filament. Due to the limited residence time of the materials in the heated nozzles and the low shear employed in FDM 3D printing [34,56], these temperatures were higher than those used for the extrusion process. The systems printed below these temperatures had problems of surface defects due to the poor melting and inconsistent flow of material through the nozzle. Other operating parameters that significantly influenced the printing process were the first layer’s speed and height, which had a major role in the adhesion of the material to the platform. For comparative purposes in the drug dissolution studies (see Section 3.8), the internal spiral compartment was also printed, being necessary a PLA support to avoid collapse during the deposition of successive layers.

**Figure 8:**
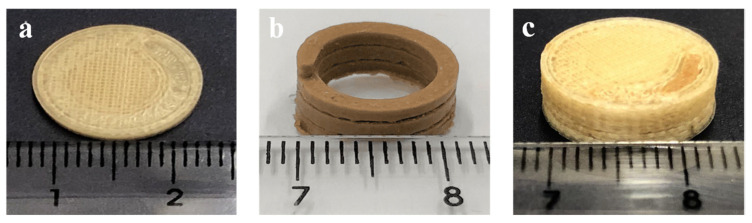
Pictures of 3D-printed devices: (**a**) cylindrical HPMCAS-based external compartment (top view), (**b**) drug-loaded PVA-based internal compartment (spiral shape), (**c**) bicompartmental device.

Due to the proper mechanical properties of the produced filaments, the 3D-printed devices were successfully printed, as can be seen in Figure 8. Both the inner compartment (spiral channel) and the bicompartmental device showed smooth and accurate structures, and are highly reproducible despite the complex internal geometry. The results of the physical characterization of the 3D-printed bicompartmental devices are presented in Table 5. Regarding their weight uniformity, the bicompartmental devices fulfill the requirements of the European Pharmacopeia [57] for tablets, with an RSD lower than 5%. The diameter and thickness of the 3D-printed bicompartmental devices also show good reproducibility and agree well with the design values (14 mm diameter and 5 mm thickness). The higher variability in the thickness in comparison to the diameter measurements could be due to some inconsistencies in the filament thickness. The 3D-printed dosage forms also showed robust mechanical strength, as breaking did not occur when the maximum force of the crushing tester was applied. As reported by other authors [27,34,43], the prominent mechanical strength of the devices was due to the performance of the printing process with 100% infill.

### 3.6. Scanning Electron Microscopy (SEM) of 3D-Printed Devices

**Figure 9:**
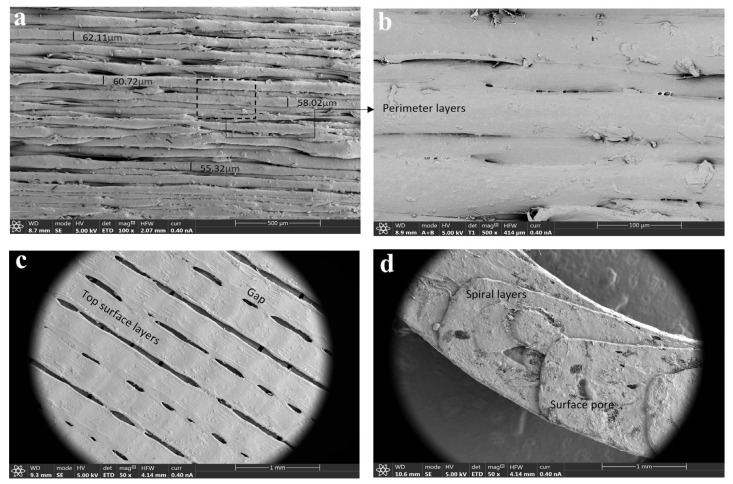
SEM microphotographs of (**a**,**b**) HPMCAS-based external compartment of the 3D-printed device (radial view, 100× and 500×); (**c**) HPMCAS-based external compartment of the 3D-printed device (axial view, 50×); (**d**) PVA-based internal compartment of the 3D-printed device (axial view, 50×).

SEM microphotographs of 3D-printed bicompartmental devices provided useful information about their external and internal structure. Radial SEM images of the HPMCAS-based external compartment (Figure 9a,b) revealed a uniform layer-by-layer deposition with a consistent layer thickness of 50–60 μm and good adhesion between them. Uniform layer deposition and close packing were also found in the literature for HPMCAS-based printed devices [21,30]. As illustrated in Figure 9c, the axial view of this compartment showed a smooth surface and a rectilinear infill pattern followed by HPMCAS-HG feedstock filament during printing. The tiny gaps or pores between the printing lines did not compromise the structural integrity of the systems. The axial view of the PVA-based internal compartment in Figure 9d demonstrates that the spiral channel was accurately and consistently printed.

### 3.7. X-ray Microcomputer Tomography (XµCT) of 3D-Printed Devices

**Figure 10:**
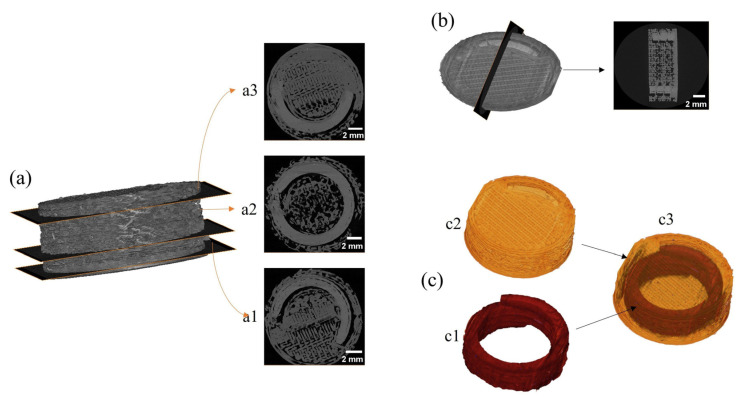
XµCT images of the 3D-printed bicompartmental device: (**a**) horizontal cross-section slices ((**a1**–**a3**), bottom to top), (**b**) vertical cross-section slice, (**c**) image segmentation and reconstruction of the 3D-printed bicompartmental device architecture ((**c1**)—inner compartment, (**c2**)—outer compartment, (**c3**)—whole structure).

XµCT analysis was also performed to investigate, in a non-destructive manner, the external and internal compartments of the 3D-printed bicompartmental devices. Figure 10a shows three horizontal cross-section slices of the 3D-printed bicompartmental device (bottom to top direction). Figure 10b displays a vertical cross-section slice at the center of the 3D-printed system. The cross-sectional images show that the spiral geometry of the inner channel was well-defined and continuous over the entire 3D-printed structure. Moreover, the grey tones in the images reveal that the PVA-based compartment (spiral shape) was denser than the HPMCAS-based external compartment.

The tomograms corresponding to the 3D-printed bicompartmental device were subjected to image segmentation processing to separate the inner compartment (red color) from the outer compartment (yellow color) (Figure 10c). As illustrated in Figure 10(c2), the external compartment presented a consistent structure, with an accurately printed top surface where the opening gap could be clearly distinguished. Homogeneous and well-fused layers were also observed at the perimeter of the external compartment, with few discontinuities that can be attributed to the slight spread of the molten printed material when deposited on top of the previous layer [15]. Figure 10(c3) displays the whole architecture of the device, making the external compartment translucent to facilitate the visualization of the spiral channel. Both compartments were clearly differentiated, demonstrating that there was no mixing or leakage of the filaments during the printing process. In addition, the spiral compartment was accurately and precisely positioned in the 3D-printed device.

As outlined by Markl et al. [58], XµCT images are extremely useful to deeply characterize the microstructural architecture of compartmental dosage forms with complex geometries.

### 3.8. In Vitro Drug Release Studies of 3D-Printed Systems

**Figure 11:**
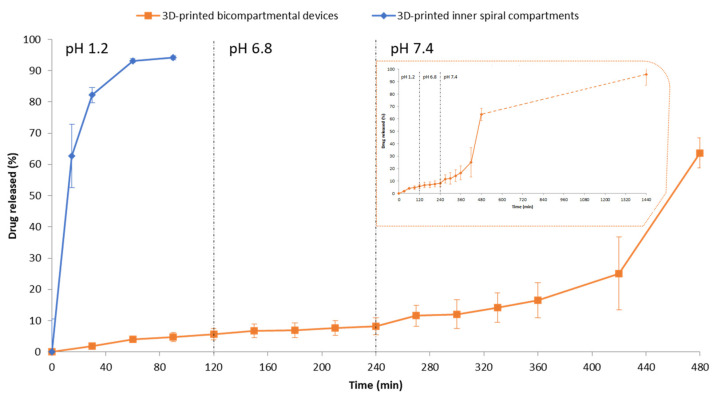
Cumulative (%) release profiles (up to 8 h) of 5-ASA from 3D-printed bicompartmental devices and 3D-printed inner spiral compartments (mean ± SD, *n* = 3). The in vitro drug release study was conducted at 37 °C in SGF pH 1.2 during the first 2 h, SIF pH 6.8 during the next 2 h, and PBS pH 7.4 until the end of the experiment. The inset in Figure 11 shows the drug release profile of 3D-printed bicompartmental devices up to 24 h.

Due to the pH-dependent solubility of HPMCAS-HG, dissolution tests were conducted using biorelevant dissolution media in a sequential pH-change method. Three-dimensional-printed bicompartmental devices obtained from HPMCAS-HG and drug-loaded PVA filaments (Figure 11) showed a biphasic drug release profile with only 5.7 wt% and 8.2 wt% drug released at pH values of 1.2 and 6.8, respectively. The low drug release in this initial phase was due to the integrity of the enteric external compartment and the slow water uptake through the PVA inner compartment. As the devices were printed with a 100% infill density, the initial sustained release phase was controlled by the water uptake rate through the opening gap, PVA solubilization, and subsequent drug diffusion. At pH 7.4, a faster release phase was obtained, increasing the cumulative drug release percentage up to 63.6 ± 4.8 wt% after 4 h in this dissolution medium, and reaching 95.9 ± 9.1 wt% at the end of the study (24 h). In agreement with the literature [26,31], the HPMCAS outer compartment, in contact with the dissolution medium, formed a viscous gel layer, dissolving at pH > 7. Then, the surface area of the PVA channel accessible to the phosphate buffer medium increased, leading to a faster release of 5-ASA.

For comparative purposes, the dissolution profile of the 3D-printed PVA-based spiral compartment was also included in Figure 11. Due to the water-solubility of PVA, a fast drug release was observed in SGF, with a cumulative drug release percentage of 94.1 ± 0.7% after 90 min. The presence of the hydrophilic plasticizer sorbitol in the PVA filament formulation also contributes to increasing the spaces between the polymeric chains, facilitating the water uptake and swelling of the PVA [37]. On the other hand, the progressive decrease in the size of the spiral compartment during the dissolution test suggests that the drug release occurred by an erosion-mediated process, in accordance with other works regarding 3D-printed PVA tablets [37,43,59].

To further elucidate the drug release mechanism of 5-ASA from the 3D-printed bicompartmental devices, the dissolution data (*M_t_*/*M_∞_* ≤ 0.6) were fitted to the Higuchi, Korsmeyer–Peppas, Peppas–Sahlin, and zero-order kinetic models. Due to the biphasic drug release profile, the release data were fitted independently for each phase. The results from mathematical modeling are collected in Table 6.

The initial drug release phases at the pH values of 1.2 and 6.8 were best fitted to the Peppas–Sahlin and Higuchi equations, followed by the Korsmeyer–Peppas and zero-order models. Although the *n* value (0.45 < *n* < 0.89) in the Korsmeyer equation is indicative of an anomalous (non-Fickian) transport, the accurate fit to the Higuchi equation and the prevalence of *k_d_* over *k_r_* in the Peppas–Sahlin model reveal that Fickian diffusion predominated over relaxation or erosion. As outlined before, this behavior agrees with the integrity of the outer enteric compartment during this phase and the slow water uptake and drug diffusion through the opening gap on top of the device. The fitting of the data to the zero-order kinetics model also confirms the slow and sustained drug release without disaggregation of the device [26,45]. At pH 7.4, a clear change in the drug release mechanism could be observed. Although the Peppas–Sahlin model provided, again, the best fit for the drug release data, the predominance of *k_r_* over *k_d_* and the poorest fit to the Higuchi equation demonstrate that drug release kinetics in this phase were mainly controlled by relaxation–erosion. Due to the solubilization of the outer HPMCAS-based compartment at pH > 7, the inner PVA-based compartment was exposed to the dissolution medium, promoting the relaxation of polymer chains and erosion and increasing the drug release rate, as observed in Figure 11. The prevalence of the relaxation–erosion mechanism is also confirmed by the *n* value higher than 0.89 in the Korsmeyer equation, indicative of a Super Case-II transport, and the poorer data fitting to the zero-order kinetics model in comparison to the initial drug release phase.

The present investigation thus demonstrates that a site-specific controlled drug release in the colonic region could be attained through the formulation and geometry design of the proposed 3D-printed bicompartmental device.

## 4. Conclusions

Through this research, bicompartmental drug delivery systems for the colon targeting of 5-ASA were successfully designed and manufactured by dual-nozzle FDM 3D printing. An analysis of the above results led to the following conclusions:

- Drug-free HPMCAS-HG and drug-loaded PVA filaments (20% *w*/*w*) were obtained by single-screw extrusion, and their suitability as feedstock materials for the 3D printing process were demonstrated through an extensive physicochemical and mechanical characterization.
- Both filaments were simultaneously printed, combining, in a single and innovative structure, an outer pH-dependent cylindrical compartment and an inner water-soluble compartment (spiral shape) containing the drug. The internal channel communicates with the surrounding media through an opening gap on top of the device. The 3D-printed bicompartmental systems showed high reproducibility despite the challenging design configuration.
- Drug release tests in biorelevant media demonstrated the ability of the novel 3D-printed structure to target 5-ASA to the colonic region. A biphasic drug release profile was obtained with an initial sustained release phase (pH values of 1.2 and 6.8) controlled by the water uptake through the opening gap, and subsequent drug diffusion from the internal PVA-based compartment (<10 wt% cumulative drug release). At pH 7.4, the solubilization of the outer HPMCAS-based compartment increased the surface area of the inner spiral exposed to the dissolution medium. Hence, the drug release kinetics changed from a diffusion-controlled to an erosion-mediated process, considerably increasing the drug release rate (>95 wt% cumulative drug release at the end of the study).

We would like to emphasize the great versatility of the designed structure, as different drug release kinetics could be obtained just by changing the dimensions of the opening gap, the solubility of the external compartment, and/or the drug distribution.

Consequently, the 3D-printed bicompartmental drug delivery system developed in this work promises to be of great help in a therapeutic field of growing interest, such as the targeted release of APIs in the colonic region, as well as to obtain extended-release devices with tunable release kinetics.

## Figures and Tables

**Table 1:** Compositions of HPMCAS and drug-loaded PVA made-in-house filaments.

Filament	Filament Composition (% *w*/*w*)			
	Polymer	Plasticizer	Drug	Lubricant/Glidant
HPMCAS	HPMCAS-HG (88%)	TEC (10%)	-	MgS (2%)
Drug-loaded PVA	PVA-MXP (51%)	Sorbitol SI 150 (27%)	5-ASA (20%)	Aerosil^®^ (2%)

**Table 2:** Printing settings for the 3D-printed bicompartmental devices and spiral compartments.

Printing Parameters	Set Values	
	Bicompartmental Device	Spiral Compartment
First layer height (mm)	0.1	0.01
Layer height (mm)	0.05	0.05
Platform temperature (°C)	60	65
First layer speed (mm/s)	10	5
Printing speed (mm/s)	40	10
Infill flowrate (%)	100	100
Infill density (%)	100	100
Infill pattern	Rectilinear	Rectilinear
Shells	2	2

**Table 3:** Extrusion process conditions and diameter values of the extruded filaments.

Filament	Temperature (°C)	Screw Speed (rpm)	Flow Speed (mm/min)	Residence Time (min)	Diameter (mm)
HPMCAS-HG	150	20 ^1^	16.4	4	1.79 ± 0.05
Drug-loaded PVA	180	30 ^2^	15.9	11	1.77 ± 0.04

^1^ Noztek Touch extruder (33 rpm, 9 Nm motor); ^2^ Noztek Touch extruder (53 rpm, 6 Nm motor).

**Table 4:** Mechanical properties of made-in-house filaments obtained by texture analysis (data represent mean ± standard deviation (*n* = 6); RSD = % relative standard deviation).

Filament	3-Point Bending Test			Stiffness Test	
	Maximum Stress (g/mm²)	Strain ^1^ at Maximum Stress (%)	Brittleness ^2^ (kg/mm²)·%	Maximum Stress (g/mm²)	Stiffness (kg/mm²)·%
HPMCAS-HG	179.22 ± 21.14 RSD 11.80	329.46 ± 39.72 RSD 12.06	55.00 ± 6.57 RSD 11.95	2870.34 ± 303.65 RSD 10.58	74.48 ± 1.95 RSD 2.62
Drug-loaded PVA	132.83 ± 10.87 RSD 8.18	355.25 ± 44.51 RSD 12.53	44.94 ± 5.51 RSD 12.25	2523.49 ± 193.82 RSD 7.68	52.86 ± 5.61 RSD 10.61

^1^ Strain (%) values in the table are equivalent to distances of 5.37 ± 0.64 mm and 6.22 ± 0.78 mm for HPMCAS-HG and drug-loaded PVA filaments, respectively. ^2^ The higher the value, the lower the brittleness.

**Table 5:** Physical characterization of the 3D-printed bicompartmental devices (data represent mean ± standard deviation (*n* = 6); RSD = % relative standard deviation).

Weight (mg)	Diameter (mm)	Thickness (mm)
811.3 ± 31.2	14.06 ± 0.07	5.09 ± 0.16
RSD 3.8	RSD 0.50	RSD 3.24

**Table 6:** Mathematical modeling and drug release kinetics of 3D-printed bicompartmental devices.

Kinetic Model	Parameters	First Phase (pH 1.2 and 6.8)	Second Phase (pH 7.4)
Higuchi (1)	*k_H_* (h^−0.5^)	0.0060	0.0334
	*r* ^2^ *_adj_*	0.9775 (*F* = 305)	0.8703 (*F* = 28)
Korsmeyer–Peppas (2)	*n*	0.67	1.78
	*k_K_* (h^−*n*^)	0.0022	0.000005
	*r* ^2^ *_adj_*	0.9495 (*F* = 133)	0.9122 (*F* = 43)
Peppas–Sahlin (3)	*k_d_* (h^−0.44^)	0.0150	−0.5252
	*k_r_* (h^−0.88^)	−0.0004	0.0222
	*r* ^2^ *_adj_*	0.9870 (*F* = 268)	0.9975 (*F* = 785)
Zero-order (4)	*k_0_* (h^−1^)	0.0003	0.0009
	*r* ^2^ *_adj_*	0.9252 (*F* = 88)	0.8963 (*F* = 36)

*k_H_*, Higuchi kinetic constant; *n*, release exponent; *k_K_*, Korsmeyer kinetic constant; *k_d_*, diffusion kinetic constant; *k*_r_, relaxation kinetic constant; *k*_0_, zero-order release rate constant; *r*^2^*_adj_*, adjusted coefficient of determination; *F*, *F* distribution for residual variance analysis (*p* < 0.01). For the Peppas–Sahlin model, *m* = 0.44 was used, as the devices under study presented an aspect ratio (diameter/thickness) of around 3.

## Data Availability

The data are contained within the article.

## Supplementary Materials

The following supporting information can be downloaded at: https://www.mdpi.com/article/10.3390/pharmaceutics15092362/s1, Figure S1: SEM microphotographs of (a) HPMCAS-HG (100×), (b) magnesium stearate (1600×), (c) physical mixture of HPMCAS-HG/TEC/magnesium stearate 88:10:2% *w*/*w* (100×), (d) Parteck^®^ MXP PVA (1200×), (e) Parteck^®^ SI 150 sorbitol (500×), (f) 5-ASA (2500×), (g) Aerosil^®^ (2500×), (h) physical mixture of PVA/sorbitol/5-ASA/Aerosil^®^ 51:27:20:2% *w*/*w* (400×).
