# Supplementary material for: Development of 3D-Printed Bicompartmental Devices by Dual-Nozzle Fused Deposition Modeling (FDM) for Colon-Specific Drug Delivery
Source: Pharmaceutics. 2023 Sep 21;15(9):2362. doi: 10.3390/pharmaceutics15092362 (PMC10535423; doi:10.3390/pharmaceutics15092362)
Supplement: Supplementary file 1 [file pharmaceutics-15-02362-s001.zip › pharmaceutics-2610157-supplementary.pdf]

<u>**SUPPLEMENTARY MATERIALS**</u>

Supplementary information (section 3.2.4. Scanning Electron Microscopy)

SEM microphotographs of the raw materials and physical mixtures are collected in Fig. S1. Granules of HPMCAS-HG (Figure S1a) presented an elongated shape with a fibrous aspect. In contrast, Magnesium stearate particles (Figure S1b) were flake-like and agglomerated due to their fine size. The microphotograph corresponding to the physical mixture (Figure S1c) showed mostly polymer particles, due to the lower proportion of the lubricant (2% w/w) in the mixture. Moreover, the morphology of the HPMCAS-HG particles seemed not to be altered by the pre-plasticization step with the liquid plasticizer TEC.

Due to the milling process of PVA Parteck® MXP, fine particles with flat and smooth surfaces could be observed (Figure S1d). Granules of Sorbitol Parteck® SI 150 were obtained by spray-drying and showed a characteristic and more spherical morphology (Figure S1e). 5-ASA was characterized by the presence of long needle or rod-like particles (Figure S1f). SEM image of Aerosil® (Figure S1g) showed fine and very light particles with a spongy structure. The microphotograph corresponding to the physical mixture (Figure S1h) confirmed that the solid-state characteristics of the materials remained unaltered after the mixing process.

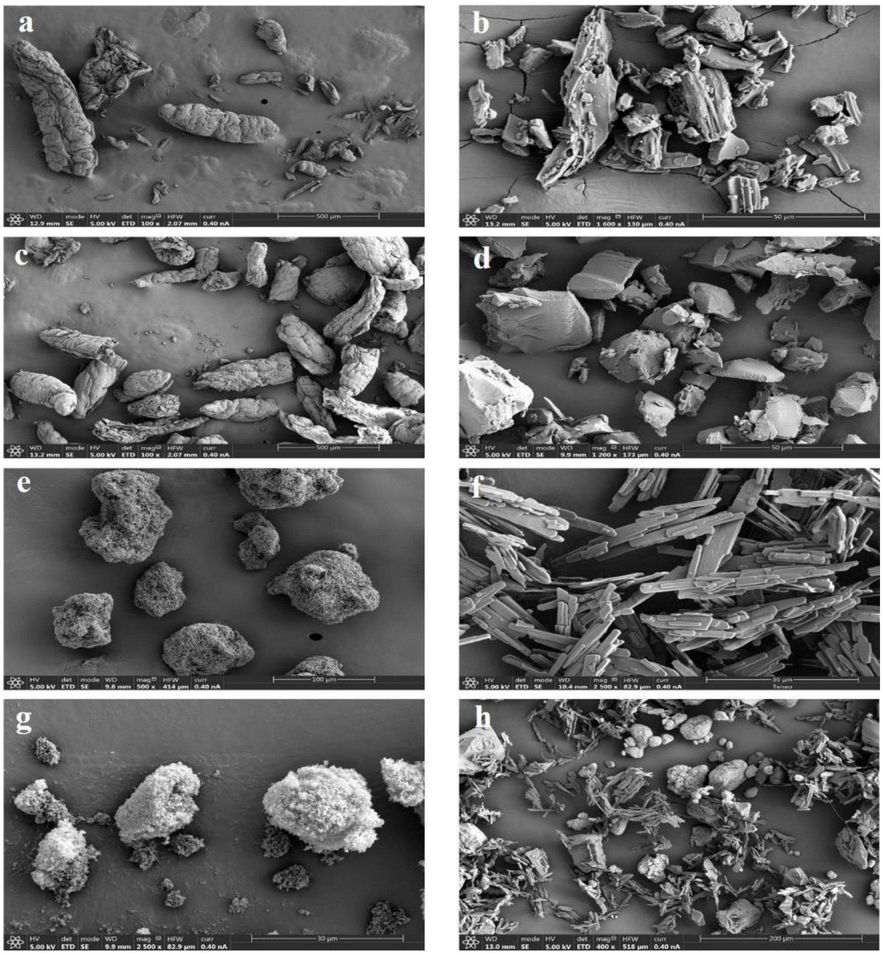

Figure S1: SEM microphotographs of (a) HPMCAS-HG (100x), (b) magnesium stearate (1600x), (c) physical mixture of HPMCAS-HG/TEC/Magnesium stearate 88:10:2% *w/w* (100x), (d) Parteck® MXP PVA (1200x), (e) Parteck® SI 150 sorbitol (500x), (f) 5-ASA (2500x), (g) Aerosil® (2500x), (h) physical mixture of PVA/Sorbitol/5-ASA/Aerosil® 51:27:20:2% *w/w* (400x).
